# Detecting copy number variation in next generation sequencing data from diagnostic gene panels

**DOI:** 10.1186/s12920-021-01059-x

**Published:** 2021-08-31

**Authors:** Ashish Kumar Singh, Maren Fridtjofsen Olsen, Liss Anne Solberg Lavik, Trine Vold, Finn Drabløs, Wenche Sjursen

**Affiliations:** 1 Department of Medical Genetics, St. Olavs Hospital, Trondheim, Norway; 2 Department of Clinical and Molecular Medicine, Faculty of Medicine and Health Sciences, NTNU - Norwegian University of Science and Technology, Trondheim, Norway

**Keywords:** Next generation sequencing (NGS), Copy number variation (CNV), Structural variant, Multiplex ligation-dependent probe amplification (MLPA), Sliding window

## Abstract

**Background:**

Detection of copy number variation (CNV) in genes associated with disease is important in genetic diagnostics, and next generation sequencing (NGS) technology provides data that can be used for CNV detection. However, CNV detection based on NGS data is in general not often used in diagnostic labs as the data analysis is challenging, especially with data from targeted gene panels. Wet lab methods like MLPA (MRC Holland) are widely used, but are expensive, time consuming and have gene-specific limitations. Our aim has been to develop a bioinformatic tool for CNV detection from NGS data in medical genetic diagnostic samples.

**Results:**

Our computational pipeline for detection of CNVs in NGS data from targeted gene panels utilizes coverage depth of the captured regions and calculates a copy number ratio score for each region. This is computed by comparing the mean coverage of the sample with the mean coverage of the same region in other samples, defined as a pool. The pipeline selects pools for comparison dynamically from previously sequenced samples, using the pool with an average coverage depth that is nearest to the one of the samples. A sliding window-based approach is used to analyze each region, where length of sliding window and sliding distance can be chosen dynamically to increase or decrease the resolution. This helps in detecting CNVs in small or partial exons. With this pipeline we have correctly identified the CNVs in 36 positive control samples, with sensitivity of 100% and specificity of 91%. We have detected whole gene level deletion/duplication, single/multi exonic level deletion/duplication, partial exonic deletion and mosaic deletion. Since its implementation in mid-2018 it has proven its diagnostic value with more than 45 CNV findings in routine tests.

**Conclusions:**

With this pipeline as part of our diagnostic practices it is now possible to detect partial, single or multi-exonic, and intragenic CNVs in all genes in our target panel. This has helped our diagnostic lab to expand the portfolio of genes where we offer CNV detection, which previously was limited by the availability of MLPA kits.

**Supplementary Information:**

The online version contains supplementary material available at 10.1186/s12920-021-01059-x.

## Background

Potentially disease-causing DNA mutations include alterations of single nucleotides up to whole chromosomes. Small changes of 1 nucleotide (nt) are called single nucleotide variation and changes up to 50 nt at single locus are called short insertion-deletion variation (indel). Whereas alterations larger than 50 nt are called structural variants (SVs) [1], which previously has been defined as alterations larger than 1000 nt [2, 3]. Such SVs include insertions, deletions, duplications, inversions, and translocations. Combinations of these SVs are also possible in a single genome [4]. Deletions and duplications, commonly called copy number variations (CNVs), contribute to a large fraction of all genetic alterations and are of diagnostic relevance as they can play important roles in causing genetic diseases [5].

Several laboratory-based approaches have been developed and can be used for detecting CNVs, including multiplex ligation-dependent probe amplification (MLPA) [6], microarray based comparative genomic hybridization (aCGH) and SNP microarrays [7], RNA sequencing [8], fluorescence in situ hybridization (FISH) [9] and PCR based methods [10]. All these methods are laboratory intensive, have low throughput and are expensive. Among these, diagnostics labs most commonly use aCGH/SNP microarray and MLPA. The aCGH method is sensitive, but it is limited to detect only CNVs of sequences present in the reference assembly used to design the array probes [11]. Limitation in MLPA-based testing is the number of probes included in the kit. It is designed to multiplex up to approximately 50 probes, hence most suitable for one or a few smaller genes.

With the evolution of next generation sequencing (NGS) technologies, diagnostics laboratories are heavily utilizing NGS data in detection of SNPs and indels. With the current quality of NGS data it is also possible to detect CNVs [12]. In addition, NGS provides the benefit of detecting exact CNV breakpoint positions in the genome. Hence using NGS for CNV detection will help diagnostic labs in testing larger number of genes for CNVs. In traditional routine diagnostic practices, samples are analyzed by MLPA testing of genes according to requests. As CNVs do not occur that often, MLPA results are often negative. It has been shown that using NGS in diagnostics provides better throughput at a lower cost compared to using MLPA-based testing for CNVs [13], and this is also consistent with the experience of our in-house diagnostic lab. MLPA is then used mainly for verification on those genes where analysis of the NGS data has indicated a CNV.

Four different approaches are currently used for detecting CNVs from NGS data [14, 15]; paired-end mapping based detection (PE), split read based detection (SR), de novo assembly based detection (DA) and read depth based detection (RD). Additionally, mixed approaches are used. All these approaches use NGS generated reads to create consensus sequences by mapping to a reference genome or by de novo assembly and looking for anomalies occurring due to SVs. Among these approaches, PE, SR and DA can be used to discover all types of SVs, but application of these approaches requires high data quality and data consistency across regions [14], which often limits their applicability to whole genome sequencing data. On the other hand, the RD approach can only detect CNVs (deletions and duplications), but it predicts exact copy numbers, including mosaicism [16, 17], and can also detect small or very large CNVs in all types of regions in a genome. Depending on data quality, coverage depth, read length, and captured regions, RD can also detect exact breakpoints with high accuracy. The best approach for CNV detection will depend upon the available sequencing data. Data from targeted gene panels represent selected genetic regions of the genome, like specific exons, which means that it does not represent continuous regions of the genome. However, as the RD approach uses region-specific information (coverage depth) to detect CNVs, this is a good approach for targeted gene panels. Due to being deep-sequenced the panel data often have high coverage depth, which increases accuracy of CNV detection via the RD approach, although the fact that intronic regions are not included in the analysis may give a somewhat lower sensitivity to certain CNVs compared to using whole genome data [18].

There are several bioinformatic tools that have been developed to detect CNVs in NGS data [13, 19]. The majority of these tools have been developed for detecting large CNVs (in the size of megabases) and hence suitable only for whole genome or whole exome sequencing data [13]. In diagnostics labs where sequencing of targeted gene panels is common practice, the main goal is often to detect small (intragenic) disease-associated CNVs in partial, single or a few small exons [20]. There are a few available tools that claim to be suitable for data from targeted gene panels [21–25], but it is always challenging to detect smaller CNVs, especially partial or single exons or mosaic CNVs, with high sensitivity and specificity consistent with diagnostic standards.

We have developed a computational pipeline to detect CNVs in NGS data from targeted gene panels, which enables us to detect small CNVs in all targets included in our panel. Since implementation of the pipeline for routine diagnostics in our lab in August 2018 it has proved its diagnostic value by detecting 45 CNVs in 16 different genes, which includes partial exonic, single exonic, multi exonic, whole gene and mosaic CNVs. By implementing this method in our routine, we have reduced cost and lab-work overhead and improved diagnostic throughput.

## Implementation

**Fig. 1:**
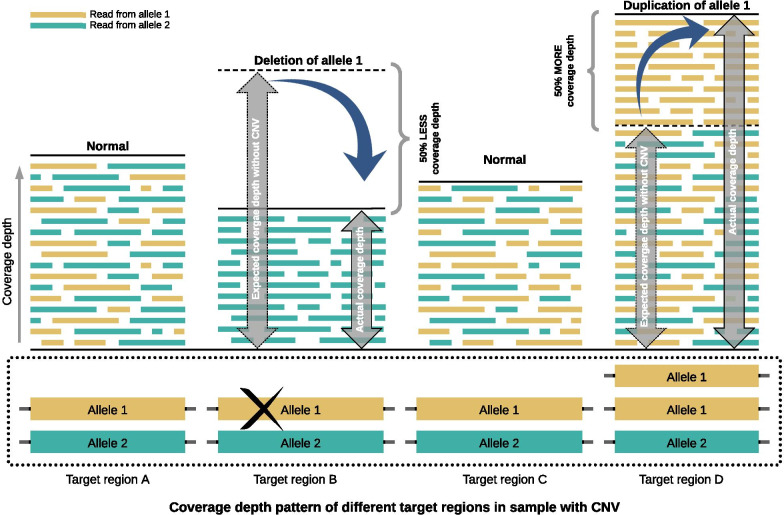
The principle of CNV detection using coverage depth information. The figure depicts the change in coverage depth of different target regions in a sample in the case of CNV events. The normal coverage depth may vary between regions, as shown for target regions A and C. It is therefore important to have access to data on normal coverage depth for each region. Deletion of allele 1 in target region B reduces the coverage in that region by 50% (i.e., to 1/2), compared to the normal or expected coverage depth for the region. Duplication of allele 1 in target region D increases the coverage in that region by 50% (i.e., to 3/2), again compared to the normal coverage depth for the region

Here we describe our CNV detection pipeline which has been developed to work on NGS data generated from targeted gene panels. To identify potential CNVs the pipeline utilizes coverage depth information of reads in target regions defined by the gene panel. If a target region has CNV, the coverage depth in this region will differ from the expected coverage depth. When duplicated, the target region will have 1.5 times more coverage depth than the expected coverage depth. On the other hand, in case of deletion the target region will have half the expected coverage depth. Figure 1 illustrates this approach of CNV detection.

**Fig. 2:**
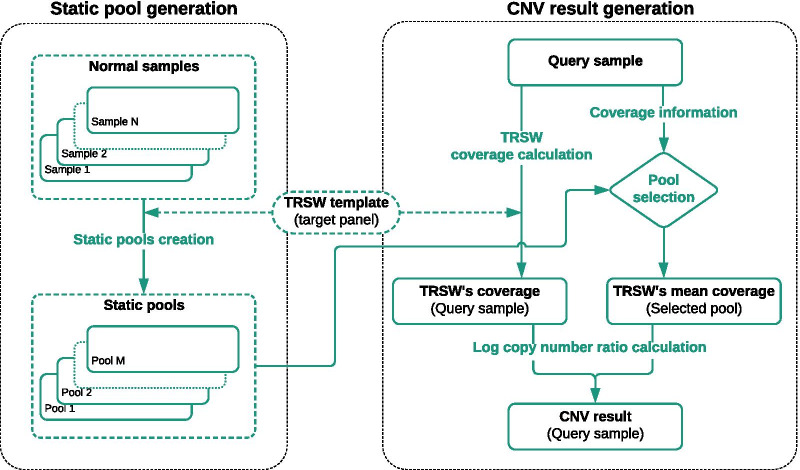
The general workflow for CNV detection. In “static pool generation” a series pools or collections of normal samples are defined to be used as reference data during CNV detection, representing the expected coverage depth of each region. In “CNV result generation” the coverage depth of the query sample is used to select a suitable pool, and a region-wise comparison between the query sample and the selected pool is used to identify regions with potential CNVs. The resolution of the comparison is improved by using a template of overlapping windows across each region of the target panel, defined as target region-based sliding windows (TRSW), see the main text for details

To detect CNVs in a target region of a query sample, our pipeline (Fig. 2) utilized this principle by comparing coverage depth in this region of the query sample with average depth in same region for normal samples with similar coverage depth as the query sample. The normal samples are provided to the analysis, and the pipeline creates pools of normal samples, where each pool contains normal samples with similar coverage depth. These pools are called static pools and can be repeatedly used for CNV detection of any query sample where the coverage depth is similar to the average coverage depth of the pool. The pipeline is illustrated in Fig. 2.

### Target region based sliding windows

**Fig. 3:**
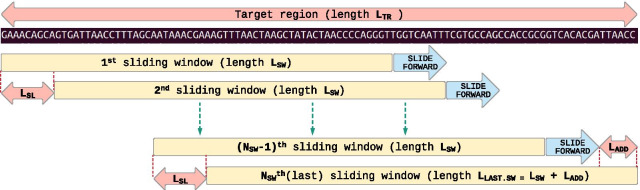
Defining the sliding window template for a target region. The selected region is divided into smaller sub-regions by a sliding window approach where each window is of a fixed size and slides forward with a fixed sliding length. The last window can be larger than the chosen window size if the length of the remaining region is smaller than sliding length. Then the remaining region is just added to the last sliding window

To increase resolution each target region is divided into overlapping sub-regions in a sliding window approach as shown in Fig. 3, forming the template for a window-based representation of each target region. This approach is called the Target Region based Sliding Windows (TRSW) approach, or just sliding windows. This also helps in detecting CNVs occurring in smaller sub-regions, e.g., part of an exon. Selection of window size is based on length of sequencing reads and the required resolution of CNV predictions. Sliding length for two adjacent overlapping sliding windows remains the same across all regions and is kept relatively small compared to window size. This helps in detecting the start- and end-points of CNVs more accurately, up to the resolution of the sliding length. At our diagnostic lab standard sequencing read length is 150 nt (X2 paired-end reads). Hence a window size of 75 nt, i.e., half of the read length, along with a sliding length of 10 nt has been chosen for validation samples and for standard routine CNV detection in NGS runs. This gives an overlap of 65 nt between two consecutive windows. This selection of window size and sliding length gives a good tradeoff between computational complexity and resolution.

Equation 1a is used for calculating N_SW_, the number of sliding windows for a target region of length L_TR_, where sliding window length is L_SW_ and sliding length is L_SL_.

1a $$N_{SW} = \frac{{L_{TR} - L_{SW} }}{{L_{SL} }} + 1$$

Window traversal for a region starts by aligning the first window at start of the region and sliding forward (with sliding length) until end of region. If for the last slide the remaining length of the region is less than sliding length, then the remaining length is added as an additional length to the last window. Hence the size of the last window in a region can be bigger than the chosen window size. Equations 1b and 1c are used for calculation of this additional length L_ADD_ and length of the last sliding window L_LAST.SW_, respectively.

1b $$L_{ADD} = \left( {L_{TR} - L_{SW} } \right)\% L_{SL}$$

1c $$L_{LAST.SW} = L_{SW} + L_{ADD}$$

Once window traversal ends for a target region, the next window starts at the beginning of the next target region. If the length of a target region is smaller than the chosen window size, then there will not be any splitting of that region into windows and there will only be one window for that region, of the same size as the region.

### Static pools from normal samples

**Fig. 4:**
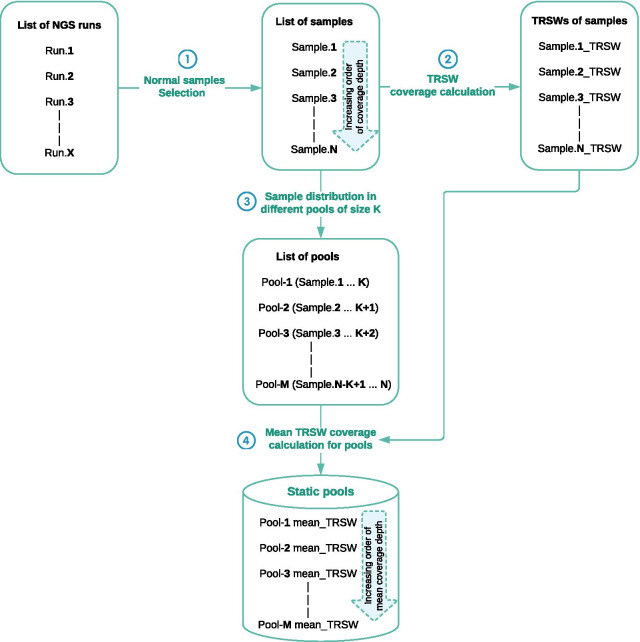
Creating static pools from normal samples. In step 1 normal samples are selected from available NGS runs and get listed in order of increasing coverage depth. In step 2 the coverage depth is calculated for each window across each sample. In step 3 the list of selected normal samples is divided into different pools of size K, where Pool-1 consists of the first K samples, followed by the next pool consisting of the next K samples after skipping the first sample of the previous pool. In step 4 the mean TRSW of each pool is calculated

In first part of the pipeline static pools are created from normal samples with no CNVs, sorted according to coverage depth. The pipeline can then select a pool of samples that matches the coverage depth of the query sample and use this to estimate expected coverage depth (without any CNVs) for a region of interest. Figure 4 shows the workflow of static pool creation.

Targeted capturing kits always have batch effects in capturing quality due to differences in batches or lots of kits as provided from vendor [26]. This is a common issue with sequencing of targeted panels. Using samples from the same sequencing batch or lot reduces the level of noise by reducing batch effects in the CNV analysis. Therefore, normal samples used in creation of static pools for a CNV analysis should be sequenced using the same batch of target capturing kit as was used for the query samples.

Results from several NGS runs are used as input data in pool creation. The pipeline extracts normal samples (with depth of coverage higher than the assigned cutoff) from the provided runs and lists them in increasing order of coverage depth (Step 1 in Fig. 4).

To increase the resolution of CNV results the sliding windows approach (TRSWs, see above) is used. For each normal sample, coverage for all sliding windows is calculated (Step 2 in Fig. 4).

This list of samples is used for creating the static pools. Equation 2 is used for calculating M, the total number of pools generated from these samples given N, the number of normal samples, and K, the pool size.

2 $$M = N - K + 1$$

Provided the size for each pool is K, the first K samples of the list are used to create the 1st static pool of normal samples, the 2nd pool skips first sample and uses the next K samples (2nd till K + 1th sample), and the same follows for next remaining pools. The Mth (last) pool uses last K samples (N − K + 1th till Nth sample) from the list (Step 3 in Fig. 4).

For each sliding window in the panel the mean coverage depth over all samples in each pool is calculated (Step 4 in Fig. 4). This list of mean coverage depth of each sliding window (mean_TRSW) of a pool is stored and used for CNV score calculations.

### CNV calculation

As all regions in the target panel are split into smaller sliding windows (TRSWs) to increase the resolution of results, CNV score is calculated for each window. Figure 2 illustrates the CNV calculation workflow.

For a given query sample the coverage depth is first calculated for each sliding window. A static pool is then chosen from the set of static pools where mean coverage depth of the selected pool is closest to coverage depth of the sample. The coverage depth for each window of the query sample is compared against mean coverage depth of each corresponding window of the selected pool. This ratio is converted to log_2_ scale to calculate the final CNV score, i.e., log copy number ratio score (logCNR score) for that window. Equation 3 is used for calculating the logCNR_score_ for a window, where L_SW_ is sliding window length, ND_i_ is nucleotide depth at ith position of query sample, ND_ij_ is nucleotide depth at ith position of jth sample in the static pool, and n is the number of samples in the selected static pool.

3 $$logCNR_{score} = log_{2} \frac{{1/L_{SW} \mathop \sum \nolimits_{i}^{{i + L_{SW} - 1}} ND_{i} }}{{1/n\mathop \sum \nolimits_{j = 1}^{n} \left( {1/L_{SW} \mathop \sum \nolimits_{i}^{{i + L_{SW} - 1}} ND_{ij} } \right)}}$$

Theoretical values of logCNR_score_ are 0.0 for 2 alleles (normal), − 1.0 for 1 allele (deletion), and + 0.58 for 3 alleles (duplication). The logCNR_score_ for each sliding window is stored as CNV results of the query sample.

### Quality control

The quality of the pools relatively to the query sample is important for the performance of our approach, and quality control of query and pools is therefore an important step for reducing noise in the analysis. Three quality checks are used. First, comparing the coverage depth of the query sample to average depth of the selected pool. Second, checking the uniformity in coverage depth among samples in the selected static pool. And third, comparing CNV results generated using static pools to results generated with run-wise pools (see below).

#### Query sample versus pool quality

Quality of CNV results depends on a similar coverage depth of query sample and selected static pool. Hence for all query samples, percentage deviation of mean depth of the query sample relative to mean depth of the selected pool is checked. If this percentage deviation is larger than a cutoff (set by lab, for example 5%), then the query sample is re-analyzed with a larger (updated) list of static pools. If the deviation is still too large, then re-sequencing or a MLPA test is used, depending on the number of genes requested for analysis.

#### Static pool quality

The quality of the selected static pool can also affect the CNV results. Even when the percentage deviation of the coverage depth of the query sample compared to mean depth of the selected pool is lower than cutoff, differences in depth of normal samples used in making of selected pools can introduce noise. Hence only good quality pools (i.e., samples with uniform coverage depth) should be used for CNV detection. Additionally, run-wise pools (created by using all samples from the same NGS run of the query sample) can also be used to check quality of the static pool in case of noisy results.

### Interpretation of output

**Fig. 5:**
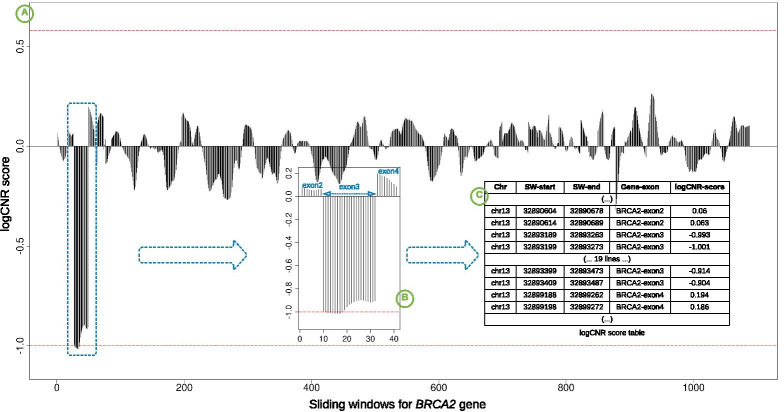
Example of output from the CNV tool. The example shows identification of a deletion of exon 3 in the BRCA2 gene in one of the control samples (CS_12). Part A shows a plot of the logCNR score for all sliding windows across the exons of the BRCA2 gene. The horizontal lines at − 1.00 and + 0.58 represent expected score values for single-allele deletions and duplications, respectively. The score values in the region of exon 3 show clear signals of a deletion. Part B shows a zoomed in representation of the plot for exon 3 with the CNV deletion, whereas its neighbouring exons (2 and 4) have normal coverage depth. Part C shows the logCNR table listing score values for selected sliding windows covering the exon2–exon3 and exon3–exon4 junctions

For each gene in the target panel, logCNR score of windows belonging to that gene are plotted. These plots are checked for initial assessment. Once potential signals are identified, gene specific regions are looked up in the table of logCNR scores. As example of a deletion event, Fig. 5 shows plots of logCNR score of all sliding windows of *BRCA2* gene in a control sample (CS_12) depicting signals of deletion of exon3, and the table in Fig. 5 enlists the logCNR scores of all sliding windows of same exon3 and its adjacent exon2 and exon4. In some cases, to get the best possible resolution (i.e., to locate exact break point) nucleotide-level coverage files are also checked. In our lab’s diagnostic practices, we also generate merged plots for the same gene across all the samples sequenced in same run (without naming the samples to avoid incidental findings), which helps in detecting or rectifying any noise or signal. We also generate merged plots for run-wise versus static pooling results for all genes over all samples, which helps us in predicting or identifying any noise associated with static pools (see Quality control).

Once CNV signals have been confirmed in the logCNR score table, MLPA-based validation in performed on the sample. In cases of specific genes where MLPA test is not available, RNA sequencing or long-range PCR is performed for CNV verification.

### Control samples

Selection of control samples for validation has been based on availability of known CNV positive samples, previously detected through MLPA. These samples were collected from the genetic diagnostic laboratories at Haukeland University Hospital (Bergen, Norway), University Hospital of North Norway (Tromsø, Norway), and St. Olavs Hospital (Trondheim, Norway). In total 36 positive control samples were used for validation of the CNV detection pipeline, where only genes with known CNVs were checked to reduce the risk of incidental findings. Additionally, 11 routine samples were chosen for calculating the specificity of the pipeline, where all the genes in the panel were checked for CNVs. These samples were collected at Department of Medical Genetics, St. Olavs Hospital, Trondheim, Norway. Both the 36 positive control samples and the 11 routine samples were germline samples where DNA had been extracted from blood.

The target gene panel consisted of 126 genes. For all genes, only exons, UTR regions and approximately ± 25 nucleotides in intronic regions were captured. These 126 genes are mainly cancer associated genes. Additional file 1 lists target regions and capturing probes.

Illumina’s Nextera Rapid Capture Custom Enrichment kit was used for capturing the target sequences. Illumina MiSeq and Illumina NextSeq 500 sequencers were used for sequencing the samples.

Among 36 positive control samples, 22 samples were sequenced once (12 on MiSeq and 10 on NextSeq sequencer), 14 samples were sequenced twice (once on MiSeq and once on NextSeq sequencer). The 11 routine samples were sequenced on MiSeq. Repetition of sequencing was performed to replicate the results, and the use of different sequencers was done to test the robustness of pipeline for differences in data quality due to different sequencing platforms.

### Data pre-processing

Sequencing data (as FASTQ files) was preprocessed to generate suitable input for the CNV pipeline, using human genome version GRCh37 [27] as the reference genome. GATK best practices guidelines [28] were used for the preprocessing, which included alignment of raw pair-end reads (FASTQ files) to the reference genome using the BWA tool [29], further sorting, marking of duplicates, INDEL realignment and base quality score recalibration steps using the GATK toolkit to generate analysis-ready aligned reads (BAM files). These aligned reads (BAM files) were used for calculating average coverage and per-locus coverage (nucleotide level coverage) of samples by using the GATK toolkit tool DepthOfCoverage. Both average coverage and per-locus coverage of samples were used as input data to the CNV pipeline for both static pool creation and CNV calculation steps.

## Results

### Validation of the pipeline

The pipeline was validated with the 36 CNV positive control samples. Only the 12 genes with known CNVs detected with MLPA or RNA sequencing were looked at. All the previously detected CNV were found with the pipeline, and comprising 4 whole gene deletions, 6 single exon deletions, 17 multi-exon deletions, 2 single + partial exon deletions (break point inside the second exon), 3 single exon duplications and 4 multi-exon duplications. Table 1 lists these 12 genes and number of findings for each of them. Additional file 2 lists the CNV findings with genomic positions.

**Table 1:** Genes with CNVs identified in positive control samples

Gene name	Type of CNV: number of findings
*APC*	Whole gene deletion:1
*BRCA1*	Single exon deletion: 2; Multi exon deletion: 6; Single exon duplication: 1
*BRCA2*	Single exon deletion: 1; Multi exon deletion: 1; Single exon duplication: 2; Whole gene deletion: 2
*CDH1*	Multi exon deletion: 1
*CDKN2A*	Single + partial^*^ exon deletion: 2
*MLH1*	Multi exon deletion: 3
*MSH2*	Single exon deletion: 1; Multi exon deletion: 4; Multi exon duplication: 1
*NF1*	Multi exon deletion: 1; Multi exon duplication: 1; Whole gene deletion: 1
*PMS2*	Multi exon duplication: 2
*PTEN*	Single exon deletion: 1
*STK11*	Single exon deletion: 1
*VHL*	Multi exon deletion: 1

^*^Here partial exon means that CNV breakpoint is inside exon

### Sensitivity, specificity and accuracy

Calculation of sensitivity for this method was based on the 36 known CNVs in the 36 positive control samples. Since all the variants were detected by the pipeline, the measured sensitivity is 100%, at least for this set of samples.

Calculation of specificity was based on the results from 11 diagnostic routine samples where all 126 genes in the target panel were checked for CNVs. In total we analyzed 1386 (11 × 126) individual genetic regions for CNVs. Of these 1386 regions, we detected 126 false positive results. This provides specificity of 90.9% and total accuracy of 91.14% for the pipeline. Additional file 3 shows details of the false positives and regions of systematic error and their respective genes in these samples.

### Using the pipeline in routine diagnostics

Since implementation of the CNV detection pipeline in routine work of our diagnostic lab in August 2018, we have detected 45 germline samples with CNVs. These CNVs were found in 16 different genes and include 5 whole gene deletions, 6 single exon deletions, 18 multi exon deletions, 1 multi + partial exon deletion, 1 multi-exon mosaic deletion, 1 whole gene duplication and 13 multi-exon duplications. Table 2 lists these 16 genes and the number of findings in each. Some of the diagnostic samples show similar CNV events, e.g., all 7 samples with CNVs in *ATM* genes, 4 out of 5 samples with CNVs in the *PMS2* gene and 7 out of 8 samples with CNVs in the *RAD51C* gene show the same duplication events. Some of these samples are from related family members. Additional file 4 lists these CNV findings with genomic positions. All these findings were verified by MLPA and/or RNA sequencing.

**Table 2:** Genes with CNVs identified in routine diagnostic samples

Gene name	Type of CNV: number of findings
*ATM*	Multi exon duplication: 7
*BRCA1*	Single exon deletion: 3; Multi exon deletion: 1; Multi + partial^*^ exon deletion: 1; Multi exon duplication: 1
*BRCA2*	Single exon deletion: 1; Multi exon deletion: 1
*CDC73*	Multi exon deletion: 1
*CDKN2A*	Whole gene deletion (homozygote): 1
*DICER1*	Single exon deletion: 2
*MLH1*	Multi exon deletion: 1
*MSH2*	Multi exon deletion: 5; Whole gene deletion: 1
*MSH6*	Whole gene duplication: 1
*NF1*	Multi exon mosaic deletion: 1 (30% mosaicism)
*NF2*	Multi exon deletion: 1
*PMS2*	Multi exon duplication: 5
*PTCH1*	Whole gene deletion: 1
*RAD51C*	Multi exon deletion: 8
*RB1*	Whole gene deletion: 1
*PTKAR1A*	Whole gene deletion: 1

^*^Here partial exon means that CNV breakpoint is inside exon

## Discussion

While keeping the needs of diagnostic labs as our central aim we have developed a CNV detection pipeline that works on NGS data from target panels. We have validated the pipeline and implemented it in routine diagnostics, and it has been used in diagnostic practices in our lab since mid-2018. Based on the experience from routine diagnostics of more than 3000 samples it has proven its diagnostic value. By using a sliding window approach to increase resolution and static pooling to reduce noise this pipeline generates high quality CNV results. With this pipeline we have detected different types of CNVs, including whole gene CNVs and CNVs occurring at exonic level, e.g., multi exonic (intra-genic), single exonic, partial exonic and mosaic CNVs. Detecting partial exonic CNVs with exact breakpoints as well as mosaic CNVs with relatively weak signals from target panel data can be challenging with available in silico methods. By being able to handle also such data this pipeline has shown its value in diagnostic use.

Validation of the pipeline was done using 36 CNV positive control samples consisting of different types of whole gene and intrageneric CNVs in 12 different genes (Table 1). The use of a larger number of positive control samples is often recommended for validation, but this was limited by the availability of known positive controls. However, by detecting all control sample CNVs, and hence giving a measured sensitivity of 100%, this pipeline meets the diagnostics requirement of no false negative results during the validation. Although we have to consider the fact that sensitivity calculation on a certain number of already known CNV positive genes may not be entirely representative of the actual performance during normal use.

The high sensitivity, specificity and accuracy of the pipeline shows that it is well suited for clinical practice. All the 126 false positive CNV detected in the 11 validation samples are in regions with very low coverage depth, which occurs due to non-optimal capturing by the capturing kit in these regions. We found 54 of these false positives to be systematic errors as these were observed consistently in the same regions in same genes across all samples. Most of these regions are homologous or repetitive regions and high GC content regions that are challenging to sequence and map. In routine practices some of these regions (often described as systematic gap regions) are tested by other methods, such as Sanger sequencing or long-range PCR. Updating the capturing kit by adding more capturing probes and modifying the target panel by removing some of the most challenging genes has over time helped our lab to improve the sequencing quality of these regions. In addition, several of the areas with systematic errors are in UTRs that are outside of the relevant analysis area, and therefore not reported to requisitioners. The analysis is therefore in practice even more specific than shown here. The calculation nevertheless provides a rough estimate of the specificity of the analysis. The 11 validation samples were chosen because no CNVs had been detected during previous analyses (MLPA) of these samples. However, not all the 126 genes were checked with MLPA in each case, which in principle can give some false negative tests, but we believe that the probability of this is very small. The number of false negatives would in any case be small, and therefore have only minor impact on the estimated specificity.

The level of systematic sequencing errors may also change when changing to a different lot of the capturing kit [26]. This can change the capturing efficiency, and hence change the quality of sequencing data. That is, a region showing systematic errors in the analysis may not have the same systematic errors when moving to a new lot. Conversely, new regions with systematic errors may also arise with the introduction of a new lot, in genes that have not previously shown such errors. To avoid this kind of batch effects, the lot number of capturing kits should therefore be changed as infrequently as possible, and a verification must always be made when introducing a new lot.

The CNV analysis may also be affected by sample properties. In in some rare cases SNPs occurring in the binding site of a probe may affect capturing of this region, and hence reduce depth. For example, in one of our routine diagnostic samples a mutation (Chr2(GRCh37):g.47643457G > A) in the middle of exon 6 (of length 134 nt) in the *MSH2* gene led to a false signal of deletion of this exon by the pipeline. This type of noise is hard to avoid but important to be aware of and consider by checking for SNPs that can affect probe binding.

The CNV analysis may also be affected by various genomic properties. Genes with repeats or with almost identical pseudogenes are always challenging for short read alignment algorithms in assigning reads to their correct genomic position, due to the ambiguity in placing a read which matches two or more identical regions. Therefore, it is challenging to estimate the correct coverage depth for such genes or regions. For example, exon 11–15 in the *PMS2* gene have duplicated sequences in the *PMS2CL* pseudogene. This can interfere with correct identification of CNVs in these regions, in most cases affecting exons 13–15 of the gene. However, we have correctly detected CNVs for this gene in all our control samples, and also detected and verified it in 5 diagnostic samples. To avoid the risk of false negatives in this gene, it always goes through MLPA test (for the whole gene) and long-range PCR test (for only exons 11–15) for CNV detection. Similarly a *SMAD4* processed pseudogene which consists of only the exonic regions of exons 2–12 of the *SMAD4* gene introduces false signals for CNVs in exons 2–12 for this gene, and not in the introns [30]. These false signals are found not only by the pipeline, but also by MLPA. However, as deletions and duplications are not restricted to exonic sequences, but should also be found in intronic regions, we can identify these CNVs as false signals introduced due to processed pseudogene.

This pipeline has now been used in our routine diagnostic practice for more than two years. Since its implementation in our diagnostics the pipeline has detected different types of challenging CNVs in 16 different genes in 45 diagnostic germline samples, as listed above (Table 2), and several of these genes were previously not tested for CNVs (with MLPA) in our diagnostic practice. This shows that the use of this pipeline has been an important expansion of our capacity for clinical diagnosis. Although most of our use so far has been on DNA extracted from blood, in a few cases the pipeline has also been used on sequencing data generated with DNA extracted from fresh frozen tissue samples. In principle the pipeline can also be used on somatic samples, and as part of our work towards future versions of the pipeline it will be tested and further developed also for the analysis of somatic samples.

Compared to some other tools our pipeline is specially designed to detect smaller CNVs in target panel-based data, e.g., single exonic and partial exonic CNVs. Splitting of larger regions into overlapping sliding windows and the possibility to choose smaller sliding length with respect to window length provides high resolution of CNV results. This improves the detection of small CNV events and predicts the variant boundaries (breakpoints) more accurately. Also, the availability of nucleotide level coverage information has facilitated prediction of exact breakpoints, especially for partial exonic CNVs. Some tools [22, 25] claim to detect CNVs at single exonic level, but it is still challenging to detect partial exonic and mosaic CNVs. Our pipeline has successfully managed to detect such CNVs in routine diagnostics, in addition to exonic CNVs.

Presently the pipeline uses a fixed window size for sliding windows across all regions in the target panel (except for last window of a region and for regions smaller than window size). As a future improvement we are considering whether the sliding window size should be chosen based on the length of each region, and the pattern of sliding windows created accordingly. This will make it possible to use larger sliding windows for larger regions, but also smaller sliding windows for smaller regions. Sliding length may also be selected according to size of the window length. This more dynamic approach can speed up the computation for larger regions, while at the same time giving sufficient resolution of CNV scores for smaller regions.

The CNV score (logCNR_score_) in our approach has a theoretical value of + 0.58 for duplications and − 1.0 for deletions. As the numeric value of the duplication score is less than the deletion score (|+ 0.58| < |− 1.0|), signals for duplications are weaker than for deletions. Interpretation of the pipeline output is based on logCNR scores and their plots, rather than a list of CNV calls. This means that no strict numerical cutoff on logCNR scores is used by our diagnostic lab. This reduces the risk of false negatives due to weak or somewhat noisy signals, and any false positives from this approach will be found by the subsequent experimental verification by sequencing or MLPA. This manual approach to output analysis is doable because most often we are asked to analyze only some of the genes included in the panel (1–15 genes), hence interpretation for this small numbers of genes can easily be managed without using strict cutoffs on CNV score. But for investigating larger sets of queries, like larger target panels with hundreds of genes, or exome panels, certain cutoffs based on statistical analysis will be necessary in order to remove most of the false positive signals caused by noise, to reduce workload and to narrow down investigation towards the most reliable CNV signals. This will be considered for future versions of our pipeline, adapted to large query sets.

To further improve the pipeline, we will in the future also update our approach to sample selection for pool creations. Presently this is based on similarity of coverage depth across samples for creating pools, for selecting pool size, and for selecting the optimal pool for a given query sample. The pattern of coverage depth in target regions remains the same across different samples sequenced from the same lot of a capturing kit. Normalization of the coverage depth of normal samples in pools and of query samples will help in creating a single pool with all available normal samples, which can be used with all query samples. This will also reduce the overhead in the pipeline in creating different pools, and in pool selection for each query sample. However, this also requires a good understanding of optimal approaches for normalization of samples and will therefore be considered mainly for future versions of our pipeline.

## Conclusions

We have here described a pipeline for detection of CNVs in NGS sequencing data from targeted gene panels. This pipeline has high sensitivity, specificity, and accuracy, and has already proven its diagnostic value with more than 45 CNV findings in routine diagnostics in our laboratory since August 2018. These findings include partial exonic, single exonic, multi exonic, whole gene and mosaic CNVs, often in genes that previously were not tested, for example because MLPA tests were not available. By using this pipeline our lab has expanded the portfolio of genes up to whole gene panels where we can offer CNV detection, which is important for the quality of our diagnostic work.

## Supplementary Information

**Additional file 1.** Target gene panel, consisting of 126 genes.

**Additional file 2.** CNV findings (with genomic positions) in 36 control samples.

**Additional file 3.** Details of false positives and regions of systematic errors and their respective genes in 11 routine samples used for quality control.

**Additional file 4.** CNV findings (with genomic positions) in 45 diagnostic routine samples.

## Data Availability

Project name: CNV detection in diagnostic gene panel. Project home page: https://github.com/ash9nov/Target-panel-based-CNV-detection. Operating system(s): Unix. Programming language: Shell Scripting, R. Other requirements: None. Licence: GNU GPLv3. Any restrictions to use by non-academics: none. Due to confidentiality and ethical concerns, data cannot be made publicly available. Further information about the data and conditions for access can be provided by the corresponding author AKS (Ashish.Kumar.Singh3@stolav.no; ashish.k.singh@ntnu.no) and by Department of Medical Genetics, St. Olavs Hospital, Trondheim (genetikk@stolav.no).

## Abbreviations

CNV: Copy number variation
CNR: Copy number ratio
TRSW: Target region based sliding windows
MLPA: Multiplex ligation-dependent probe amplification
NGS: Next generation sequencing
PE: Paired-end mapping based detection
SR: Split read based detection
DA: De novo assembly based detection
RD: Read depth based detection
SV: Structural variant
aCGH: Array comparative genomic hybridization
SNP: Single nucleotide polymorphism
PCR: Polymerase chain reaction
FISH: Fluorescence in situ hybridization
UTR: Untranslated region
