# Supplementary material for: Detecting copy number variation in next generation sequencing data from diagnostic gene panels
Source: BMC Med Genomics. 2021 Aug 31;14:214. doi: 10.1186/s12920-021-01059-x (PMC8406611; doi:10.1186/s12920-021-01059-x)
Supplement: Supplementary file 2 — Additional file 2. CNV findings (with genomic positions) in 36 control samples. [file 12920_2021_1059_MOESM2_ESM.docx]

**Table S1: List of 36 CNV findings in positive control samples
(*del: deletion; dup: duplication; ex: exon)**

| **Sample ID** | **Gene** | **CNV** | **g.DNA position (GRCh37)** | **c.DNA position** |
| --- | --- | --- | --- | --- |
| CS_1 | APC | Whole gene deletion | chr5:112043177-112181961 | NM_000038.4 (c.-30464 - c.*2138) |
| CS_2 | BRCA1 | del ex 7-13 | chr17:41231326-41251922 | NM_007294.3 (c.4423+25 - c.442-25) |
| CS_3 | BRCA1 | del ex1-13 | chr17:41234396-41277525 | NM_007294.3 (c.-257 - c.4357+25) |
| CS_4 | BRCA1 | del ex16 | chr17:41222920-41223280 | NM_007294.3 (c.4676-25 - c.4986+25) |
| CS_5 | BRCA1 | del ex18-24 | chr17:41196287-41215993 | NM_007294.3 (c.5075-25 - c.*1408) |
| CS_6 | BRCA1 | del ex24 | chr17:41196287-41197844 | NM_007294.3 (c.5468-25 - c.*1408) |
| CS_7 | BRCA1 | del ex3-16 | chr17:41222920-41277525 | NM_007294.3 (c.81-25 - c. 4986+25) |
| CS_8 | BRCA1 | del ex5-7 | chr17:41256114-41258575 | NM_007294.3 (c.135-25 - c.441+25) |
| CS_9 | BRCA1 | del ex8-13 | chr17:41231326-41251922 | NM_007294.3 (c. 442-25 - c.4358-2695) |
| CS_10 | BRCA1 | dup ex13 | chr17:41231326-41234617 | NM_007294.3 (c.4186-25 - c.4358-2695) |
| CS_11 | BRCA2 | del ex 19-21 | chr13:32944514-32950953 | NM_000059.3 (c.8332-25 - c.8754+25) |
| CS_12 | BRCA2 | del ex3 | chr13:32893189-32893487 | NM_000059.3 (c.68-25 - c.316+25) |
| CS_13 | BRCA2 | dup ex20 | chr13:32945068-32945262 | NM_000059.3 (c.8488-25 - c.8632+25) |
| CS_14 | BRCA2 | dup ex20 | chr13:32945068-32945262 | NM_000059.3 (c.8488-25 - c.8632+25) |
| CS_15 | BRCA2 | Whole gene deletion | chr13:32889592-32973834 | NM_000059.3 (c.-252 -c.*927) |
| CS_16 | BRCA2 | Whole gene deletion | chr13:32889592-32973834 | NM_000059.3 (c.-252 - c.*927) |
| CS_17 | CDH1 | del ex11-13 | chr16:68853158-68857554 | NM_004360.3 (c.1566-25 -c.2164+25) |
| CS_18 | CDKN2A | Deletion of parts of 5` flanking region, ex 1, intron 1 and parts of ex 2 | chr9:21971045-21975157 | NM_000077.4 (c.-331 - c.313) NM_058195.3(c.194-3950 - c..356) |
| CS_19 | CDKN2A | Deletion of parts of 5` flanking region, ex 1, intron 1 and parts of ex 2 | chr9:21971045-21975157 | NM_000077.4 (c.-19689 – c.313) NM_058195.3(c.-185 - c.356) |
| CS_20 | MLH1 | del ex 7-9 | chr3:37053286-37056060 | NM_000249.3 (c.546-25 - c.790+25) |
| CS_21 | MLH1 | del ex11-12 | chr3:37061776-37067523 | NM_000249.3 (c.885-25 - c.1409+25) |
| CS_22 | MLH1 | del ex7-9. | chr3:37053286-37056060 | NM_000249.3 (c.546-25 - c.790+25) |
| CS_23 | MSH2 | del ex 2-7 | chr2:47635515-47657105 | NM_000251.2 (c.212-25 - c.1276+25) |
| CS_24 | MSH2 | del ex1 | chr2:47630181-47630566 | NM_000251.2 (c.-150 - c.211+25) |
| CS_25 | MSH2 | del ex1-6 | chr2:47630181-47643593 | NM_000251.2 (c.-150 - c.1076+25) |
| CS_26 | MSH2 | del ex2-7 | chr2:47635515-47657105 | NM_000251.2 (c.212-25 - c.1276+25) |
| CS_27 | MSH2 | del ex4-7 | chr2:47639528-47657105 | NM_000251.2 (c.646-25 - c.1276+25) |
| CS_28 | MSH2 | dup ex 3-15 | chr2:47637208-47708035 | NM_000251.2 (c.367-25 - c.2634+25) |
| CS_29 | NF1 | del ex5-51. | chr17:29496884-29679457 | NM_001042492.2 (c.480-25 - c.7615+25) |
| CS_30 | NF1 | dup ex25-37. | chr17:29559066-29653295 | NM_001042492.2 (c.3198-25 - c.5268+25) |
| CS_31 | NF1 | Whole gene deletion | chr17:29421920-29704720 | NM_001042492.2 (c.-408 - c.*3548) |
| CS_32 | PMS2 | dup ex 11-12 | chr7:6022430-6027276 | NM_000535.5 (c.1145-25 - c.2174+25) |
| CS_33 | PMS2 | dup ex11-12 | chr7:6022430-6027276 | NM_000535.5 (c.1145-25 - c.2174+25) |
| CS_34 | PTEN | del ex 8 | chr10:89720626-89720900 | NM_000314.4 (c.802-25 - c.1026+25 |
| CS_35 | STK11 | del ex1 | chr19:1205773-1207227 | NM_000455.4 (c.-1140 - c.290+25) |
| CS_36 | VHL | del ex2-3 | chr3:10188173-10192810 | NM_000551.3 (c.341-25 - c.*1161) |

Detection of exact breakpoint inside exon-2 (Samples CS_18 & CS_19) was done by comparing the sample coverage with mean coverage of static pool at nucleotide level. Also, coverage depth change patterns were compared at breakpoint for these samples against samples sequenced in same NGS run.
