# Supplementary material for: Detecting copy number variation in next generation sequencing data from diagnostic gene panels
Source: BMC Med Genomics. 2021 Aug 31;14:214. doi: 10.1186/s12920-021-01059-x (PMC8406611; doi:10.1186/s12920-021-01059-x)
Supplement: Supplementary file 4 — Additional file 4. CNV findings (with genomic positions) in 45 diagnostic routine samples. [file 12920_2021_1059_MOESM4_ESM.docx]

**Table S2: List of 45 CNVs findings in samples from routine diagnostics**

| Sample ID | Gene | CNV | g.DNA position (GRCh37) | c.DNA position |
| --- | --- | --- | --- | --- |
| RD_1 | ATM | ex62-3'UTRdup | g.108235784_g.108239710dup | NM_000051.3 (c.8851-25_c.*3475dup) |
| RD_2 | ATM | ex62-3'UTRdup | g.108235784_g.108239710dup | NM_000051.3 (c.8851-25_c.*3475dup) |
| RD_3 | ATM | ex62-3'UTRdup | g.108235784_g.108239710dup | NM_000051.3 (c.8851-25_c.*3475dup) |
| RD_4 | ATM | ex62-3'UTRdup | g.108235784_g.108239710dup | NM_000051.3 (c.8851-25_c.*3475dup) |
| RD_5 | ATM | ex62-3'UTRdup | g.108235784_g.108239710dup | NM_000051.3 (c.8851-25_c.*3475dup) |
| RD_6 | ATM | ex62-3'UTRdup | g.108235784_g.108239710dup | NM_000051.3 (c.8851-25_c.*3475dup) |
| RD_7 | ATM | ex62-3'UTRdup | g.108235784_g.108239710dup | NM_000051.3 (c.8851-25_c.*3475dup) |
| RD_8 | BRCA1 | 5'UTR-ex12del | g.41277500_g.41234396del | NM_007294.3 (c.-232_c.4357+25del) |
| RD_9 | BRCA1 | ex7del | g.41251922_g.41251767del | NM_007294.3 (c.442-25_547+25del) |
| RD_10 | BRCA1 | ex15del | g.41223280_g.41222920del | NM_007294.3 (c.4676-25_c.4986+25del) |
| RD_11 | BRCA1 | 5'UTR-ex3dup | g.41277500_g.41267718dup | NM_007294.3 (c.-232_c.134+25dup) |
| RD_12 | BRCA1 | ex4-5-half-ex6del | g.41258575_g.41256206del | NM_007294.3 (c.135-25_c.374del) |
| RD_13 | BRCA1 | ex15del | g.41223280_g.41222920del | NM_007294.3 (c.4676-25_c.4986+25del) |
| RD_14 | BRCA2 | ex22-24del | g.32953429_g.32954307del | NM_000059.3 (c.8755-25_c.9256+25del ) |
| RD_15 | BRCA2 | ex25del | g.32968801_g.32969095del | NM_000059.3 (c.9257-25_c.9501+25del) |
| RD_16 | CDC73 | ex2-10del | g.193094217_g.193121599del | NM_024529.4 (c.132-25_c.972+25del) |
| RD_17 | CDKN2A | Whole gene deletion | g.21975132_g.21967751del | NM_000077.4 (c.-306_c.*477del) NM_058195.3 (c.194-3925_*592del) |
| RD_18 | DICER1 | ex8del | g.95591030_g.95590508del | NM_177438.2 (c.904-25_1376+25del) |
| RD_19 | DICER1 | ex8del | g.95591030_g.95590508del | NM_177438.2 (c.904-25_1376+25del) |
| RD_20 | MLH1 | ex7-9del | g.37053286_g.37056060del | NM_000249.3 (c.546-25_c.790+25) |
| RD_21 | MSH2 | ex2-7del | g.47635515_g.47657105del | NM_000251.2 (c.212-25_c.366+25del) |
| RD_22 | MSH2 | ex7-8del | g.47656856_g.47672821del | NM_000251.2 (c.1077-25_c.1386+25del) |
| RD_23 | MSH2 | Whole gene deletion | g.47630206_g.47710367del | NM_000251.2 (c.-125_c.*279del) |
| RD_24 | MSH2 | ex2-7del | g.47635515_g.47657105del | NM_000251.2 (c.212-25_c.366+25del) |
| RD_25 | MSH2 | ex2-7del | g.47635515_g.47657105del | NM_000251.2 (c.212-25_c.366+25del) |
| RD_26 | MSH2 | ex2-7del | g.47635515_g.47657105del | NM_000251.2 (c.212-25_c.366+25del) |
| RD_27 | MSH6 | Whole gene duplication | g.48010221_g.48034092dup | NM_000179.2 (c.-152_c.*93dup) |
| RD_28 | NF1 | 5'UTR-ex11**Mosaic** del | g.29421945_g.29528528del | NM_001042492.2 (c.-383_c.1260+25del) |
| RD_29 | NF2 | 5’UTR_ex1del | g.29999520_ 30000126del | NM_000268.3 (c.-468_c.114+25del) |
| RD_30 | PMS2 | ex11-3'UTRdup | g.6027276_g.6012870dup | NM_000535.5 (c.1145-25_c.*160dup) |
| RD_31 | PMS2 | ex11-12dup | g.6027276_g.6022430dup | NM_000535.5 (c.1145-25_c.2174+25dup) |
| RD_32 | PMS2 | ex11-3'UTRdup | g.6027276_g.6012870dup | NM_000535.5 (c.1145-25_c.*160dup) |
| RD_33 | PMS2 | ex11-3'UTRdup | g.6027276_g.6012870dup | NM_000535.5 (c.1145-25_c.*160dup) |
| RD_34 | PMS2 | ex11-3'UTRdup | g.6027276_g.6012870dup | NM_000535.5 (c.1145-25_c.*160dup) |
| RD_35 | PTCH1 | Whole gene deletion | g.98270831_g.98205264del | NM_000264.3 (c.-188_c.*3411del) |
| RD_36 | PTKAR1A | Whole gene deletion | g.66507896_g.66529595del | NM_002734.4 (c.-800_c.*3005del) |
| RD_37 | RAD51C | ex4-3'UTRdel | g.56780532_g.56811692del | NM_058216.1 (c.572-25_c.*109del) |
| RD_38 | RAD51C | ex4-3'UTRdel | g.56780532_g.56811692del | NM_058216.1 (c.572-25_c.*109del) |
| RD_39 | RAD51C | ex4-3'UTRdel | g.56780532_g.56811692del | NM_058216.1 (c.572-25_c.*109del) |
| RD_40 | RAD51C | ex5-3'UTRdel | g.56787195_g.56811692del | NM_058216.1 (c.706-25_c.*109del) |
| RD_41 | RAD51C | ex4-3'UTRdel | g.56780532_g.56811692del | NM_058216.1 (c.572-25_c.*109del) |
| RD_42 | RAD51C | ex4-3'UTRdel | g.56780532_g.56811692del | NM_058216.1 (c.572-25_c.*109del) |
| RD_43 | RAD51C | ex4-3'UTRdel | g.56780532_g.56811692del | NM_058216.1 (c.572-25_c.*109del) |
| RD_44 | RAD51C | ex4-3'UTRdel | g.56780532_g.56811692del | NM_058216.1 (c.572-25_c.*109del) |
| RD_45 | RB1 | Whole gene deletion | g.48877883_g.49056026del | NM_000321.2 (c.-166_c.*1819del) |
